# Predictors of Self-Medication for Sleep Disturbances in Tunisia: A Cross-Sectional Study

**DOI:** 10.1192/j.eurpsy.2026.12095

**Published:** 2026-07-31

**Authors:** K. Mahfoudh, S. Walha, S. Hamzaoui, M. Mrad, R. Hosni, R. Hosni, A. Ouertani, A. Aissa, R. Jomli

**Affiliations:** Avicenne, Razi Hospital, Manouba, Tunisia

## Abstract

**Introduction:**

Self-medication for sleep disturbances is a growing public health concern, particularly in the absence of medical supervision. Identifying the factors associated with this behavior is essential to guide targeted prevention strategies and promote safer sleep health management.

**Objectives:**

To explore sociodemographic, clinical, behavioral, and sleep-related factors associated with self-medication for sleep disorders in a sample of the general Tunisian population.

**Methods:**

We conducted a descriptive and analytical cross-sectional study using an anonymous online questionnaire disseminated via social media platforms. The study targeted Tunisian adults aged 18 to 65. Data collected included sociodemographic characteristics (age, gender, marital status, number of children, educational level, occupation, place of residence, and work schedules), clinical history (including psychiatric and somatic conditions), sleep-related variables (using the Pittsburgh Sleep Quality Index - PSQI), lifestyle habits, perceived stress, and self-medication practices. Data were analyzed using SPSS, and descriptive and inferential statistics were applied.

**Results:**

We included 287 participants, mostly women (61%) and young adults (53% aged 26–35). The majority lived in urban areas (93%) and 21% reported psychiatric history. Stress levels were moderate to high in most participants.

The average PSQI score was 11.55 ± 3.5, with 33.79% of participants indicating sleep disturbances.

Bivariate analysis showed that female gender, psychiatric history, psychiatric treatment, higher stress, higher PSQI scores, longer sleep problems, and medical consultation were significantly associated with self-medication. Adverse effects of non-prescribed hypnotics and professional advice on risks were also linked to higher self-medication, while physical activity was protective.

Multivariate analysis revealed two independent predictors of self-medication: experiencing adverse effects from hypnotics (B=1.818; p<0.001) and receiving professional advice about risks (B=0.746; p=0.039).

**Conclusions:**

Self-medication for sleep disorders in Tunisia is driven by a complex interplay of psychological, clinical, and informational factors. The association with psychiatric history, perceived stress, and even prior medical advice highlights a gap in patient care and communication. Addressing these factors through clearer guidance and better mental health support is essential to reduce reliance on unsupervised treatments and promote safer sleep practices.

**Disclosure of Interest:**

None Declared

